# Don’t go chasing waterfalls: Multiple factor prediction of injuries in a performance context

**DOI:** 10.1016/j.jsampl.2025.100097

**Published:** 2025-04-08

**Authors:** Melanie I. Stuckey, Dean Kriellaars

**Affiliations:** Centre de recherche sur le potential humain, École Nationale de Cirque, 8181 Ave du Cirque, Montréal, Québec, H1Z 4N9, Canada

**Keywords:** Athletic injury, Logistic models, Risk factors, Risk management, Circus, Performing arts

## Abstract

Injury surveillance informs risk management strategies for athletes and performing artists yet the predictive value of multiple factors is understudied. This study analyzed demographic, sleep, fatigue, body composition, symmetry/proportionality, and psychological data from a cohort of circus arts students to predict injury presence and duration using regression models. Demographics alone significantly predicted injury presence (R^2^ ​= ​0.344) and duration (R^2^ ​= ​0.247). Additional factors explained 1.5–9.0 ​% of variation for injury presence and 0.0–7.8 ​% for duration. These findings inform the development of holistic risk management strategies for performing artists and athletes and cautions against chasing single predictor variables.

## 1 Introduction

Injury surveillance informs resource allocation and strategies to support the wellbeing of athletes and performing artists [1,2]. Injury rates and characteristics have been reported in professionalizing circus schools [[3], [4], [5], [6], [7]], but investigation of associated factors has not been undertaken. In other circus contexts (amateur, pre-professional, and professional circus artists), physical, psychosocial, and environmental factors have been associated with accidents, near misses, and injuries [[8], [9], [10]]. Similar to sport contexts [11], these studies used only a single category of predictive factors, and none combined physical, psychosocial and demographic factors. Lastly, previous studies are limited to prediction of injury presence. The International Olympic Committee suggested that other injury metrics, such as duration and burden, should be considered for elucidation of factors related to injuries [1,2]. This study explored the prediction of injury presence and duration in students in a professionalizing circus college using a reflective modelling approach based on multiple modifiable and non-modifiable predictors including demographic, physical, physiological, and psychological parameters.

## 2 Methods

This cohort study examined the prediction of injury in student circus artists enrolled in a three-year professional college training program (National Circus School, Montreal) with characteristics of the program previously described [6]. All students were eligible for inclusion in the study, and those recruited provided written, informed consent. This study was approved by the University of Manitoba Health Research Ethics Board (H2016).

Participants' demographics included age, sex at birth, year in the program (years 1–3, and year “0” corresponding to a preparatory year), and category of circus family disciplines (ground acrobatics, aerial acrobatics, equilibrium (balance), manipulation (juggling) and clowning). The predictive factors (sleep, fatigue, body composition, symmetry, psychological) were determined by a concurrent study of performance demands in the professionalizing program [[12], [13], [14]]. Participants also completed two surveys on electronic tablets to assess: 1) sleep (duration, quality, latency) and fatigue (wakefulness, daytime fatigue) with a partially validated 7-day self-report recall survey, slightly modified for the circus context [12]; and 2) psychological parameters including state anxiety, anxiety management, coping ability, perceived access to coping resources, and daily hassles [13]. Body mass index was derived from participant height (m) and mass (kg). A multi-frequency bioelectrical impedance device (mfBIA, InBody 230, InBody Co., Ltd.) was used to assess fat mass, lean body mass, and skeletal muscle mass relative to total body mass [14]. Symmetry indices for the upper and the lower limbs were derived as the absolute value of the difference in mass from right to left expressed as a percentage of body mass. Proportionality indices were calculated relating the lower and upper limb masses to trunk and total body mass. Summed predictor scores were created for each variable set with inverse scales inverted. Injury data were extracted from a research injury database derived from administrative data from an in-situ therapy clinic [6]. All injuries treated by the school’s athletic therapists were categorized based on the occurrence (presence or absence) and duration (no injury, short ≤ 4 weeks, long >4 weeks, similar to studies using treatment duration rather than time loss [6]).

Binomial and multinomial logistic regression were deployed for the dichotomous injury presence variable and the three level injury duration variable, respectively. Regression was performed for demographic data alone, then predictive models were generated using demographics in combination with sleep, fatigue, body composition, symmetry/proportionality, and psychological indices for prediction of both injury outcomes. The overall model fit was derived (Chi square, p-value) along with the coefficient of determination (R^2^) using Nagelkerke’s adaptation. For demographics predictor variables, the contribution of each variable was assessed using Likelihood Ratio Omnibus test with Odds ratios. Multicollinearity checks using variance inflation factor (threshold ​= ​5) revealed no suprathreshold collinearity among variables.

## 3 Results

Students enrolled in the professional circus arts program (*n* ​= ​93, 92 ​% of cohort) were recruited with 20, 25, 20, and 28 from each year (0–3), respectively. The circus family disciplines represented were; acrobats (33.3 ​%), aerialists (40.7 ​%), equilibrium (12.9 ​%), manipulation (10.8 ​%), and clowning (2.2 ​%). Male students (*n* ​= ​60) were on average (SD) 20.6 (0.4) years, 174.2 (5.8) cm, 72.2 (9.6) kg. Female students (*n* ​= ​33) were 20.0 (0.3) years, 162.4 (5.6) cm, 56.3 (4.4) kg.

A total of 162 injuries were documented. Of the injured cohort (*n* ​= ​59), 41 individuals (69.5 ​%) had short duration injuries (≤4 weeks) and 18 (30.5 ​%) had long duration injuries (>4 weeks). The logistic regression based on general demographics to predict the presence of injury and duration of injuries are shown in Table 1. Regressions for demographics in combination with each suite of predictor variables (sleep, fatigue, body composition, symmetry/proportionality, and psychological) are shown in Table 2.

**Table 1:** Logistic regression for general demographics to predict the presence and duration of injuries.

	Presence^A^	Duration^B^
Nagelkerke R^2^	0.344	0.247
X^2^	25.1	34.5
P	0.003	0.011
Demographics variables∗		
Sex	NS	NS
Year in program	<0.001	0.007
Age	NS	NS
Circus discipline	0.055	0.045

NS – not significant.

A Binomial logistic regression (uninjured, injured).

B Multinomial logistic regression (uninjured, ≤4 weeks, >4 weeks).

∗ Likelihood Ratio Test for each predictor variable.

**Table 2:** Regression outcomes for demographics plus each suite of predictor variables (sleep, fatigue, body composition, symmetry and psychological). Significant predictions would arise from reductions in injuries associated with improvements in composite scores.

		Presence^A^			Duration^B^		
Predictor variables sets		R^2^	P value	Additive prediction (%)	R^2^	P value	Additive prediction (%)
Demographics alone		0.344	0.003	–	0.247	0.011	–
Demographics	+ Sleep	0.359	0.042	1.5	0.266	NS	–
	+ Fatigue	0.414	0.011	7.0	0.310	0.044	6.3
	+ Body composition	0.401	0.007	5.7	0.323	0.017	7.6
	+ Symmetry/proportionality	0.432	0.008	8.8	0.325	0.048	7.8
	+ Psychological	0.434	0.003	9.0	0.279	0.011	3.2

NS – not significant.

Don’t go chasing waterfalls: multiple factor prediction of injuries in a performance context.

A Binomial logistic regression (uninjured, injured).

B Multinomial logistic regression (uninjured, ≤4 weeks, >4 weeks).

## 4 Discussion

Previous studies have shown that demographic, physical, and psychosocial factors predict injury presence [8,9], accidents and near misses [10] in circus. This study demonstrated that sleep, fatigue, body composition, symmetry/proportionality, and psychological factors provided modest additive value to the demographic prediction of injury presence in a professional circus college. Except for sleep, these parameters also modestly enhanced the demographic prediction of injury duration. Notably, the added predictive value varied between injury parameters, underscoring the need for further research on injury burden and other injury characterizations in performance contexts [1,2,6].

Injury risk management frameworks are required for performance optimization, yet often adopt a linear causation or formative approach, assuming parameters linked to injury are direct risk factors [15]. This often leads researchers and practitioners to “chase the predictor” without consideration of the complexity of the problem, including the dynamic, recursive nature of risk factors [16] and their interdependence [15]. While this study identified modifiable parameters associated with injury, we caution against a reductionist approach to resource allocation. Selecting the most relevant injury predictive parameter and focusing on those with the highest predictive value ignores the interplay between factors (e.g., sleep and anxiety), the dynamic nature of training and injury risk, including carryover effects from prior injuries, and the potential of attributing injury to personal traits rather than social biases.

Although age and sex were not significant predictors individually, non-modifiable demographic factors (age, sex at birth, year in the program, and circus family) predicted 34.4 ​% of injury presence and 24.7 ​% of injury duration. Previous studies on circus populations have yielded mixed results regarding demographics and injury or accident occurrence [3,[6], [7], [8],^10^]. No prior studies have examined the association of demographics with injury duration, though one study graphically depicted differences in injury burden by circus discipline [6]. Although demographic factors are non-modifiable, related social factors might be addressed. In sport, for example, ACL injuries are more common in females, which can potentially be attributed more to gender constructs rather than biological sex differences [17]. Mitigating gender bias in training could reduce these disparities. Research examining the effects of injury mitigation interventions targeting gender bias in circus and sport is needed.

The physical characteristics, body composition and symmetry/proportionality explained an additional 5.7 and 8.8 ​% of the variance in injury presence and 7.6 and 7.8 ​% in injury duration, respectively, beyond demographic factors, which has implications for physical preparation. Training for circus and sport often creates asymmetries or imbalances, which, if excessive, may contribute to injury. However, it’s important to discern whether these imbalances indicate injury vulnerability or simply reflect the body types typical of certain circus disciplines associated with higher injury rates. Similar to playing different positions in sport, certain body types tend to be typical of each circus discipline. Since this study and others have shown circus family or discipline to be predictive of injury or accidents [6,8,10], this needs to be elucidated in future research. This distinction between formative and reflective modeling approaches to injuries is crucial for designing effective screening and conditioning programs, as well as crucial for effective resource allocation for injury prevention. Including quaternary prevention strategies may better address individual risk management, as over-medicalizing apparent imbalances without considering their relevance to the activity and potential confounders could lead to ineffective interventions [18].

Sleep slightly improved the model’s explained variance for injury presence, while fatigue and psychological factors moderately enhanced predictions for both injury presence and duration. Psychological factors have been linked to injuries [9] or accidents and near misses [10] in circus artists, and sleep has been associated with injuries in sport [11,19]. It is likely that complex inter-relationships exist among many of these variables and others [10]; specifically sleep, fatigue, coping, challenges, and anxiety [20]. We addressed potential collinearity with assumption checks to reject correlated items. Considering both the interdependence of parameters and individual differences (e.g. sleep challenges caused by physiological vs. environmental factors), interventions for injury prevention should adopt a holistic, personalized approach rather than targeting single factors like sleep hygiene or anxiety management. Moving from population-based strategies to cluster or individualized approaches could enhance injury risk management [15].

This study has limitations. Although these data were collected prospectively, the small sample size of just over 90 participants limits the detection of weaker associations and increases the risk of chance findings. Future research should consider additional factors, such as self-efficacy [9], alongside the factors measured in this study and others.

## 5 Conclusion

When demographics were combined individually with sleep, fatigue, body composition, symmetry/proportionality, and psychological factors improved predictions of injury presence and duration compared to demographics alone. These findings demonstrated the value in using multiple predictors for injuries characterization and the need to further understand nuanced relationships among them. This study provided insights for allocation of resources and deriving interventions for risk management strategies so as to avoid chasing single predictor variables.

## Declaration of generative AI and AI-assisted technologies in the writing process

During the final stages of preparation of this work the authors used ChatGPT to shorten the discussion. After using this tool/service, the authors reviewed and edited the content as needed and take full responsibility for the content of the publication.

## Funding

Funding provided by the Social Sciences and Humanities Research Council for the Industrial Research Chair in Circus Arts (Patrice Aubertin).

## Declaration of competing interest

The authors have no competing interests to disclose.
